# Application of Metabolomics in the Study of Natural Products

**DOI:** 10.1007/s13659-018-0175-9

**Published:** 2018-06-29

**Authors:** Qi Zhao, Jia-Le Zhang, Fei Li

**Affiliations:** 1 State Key Laboratory of Phytochemistry and Plant Resources in West China, Kunming Institute of Botany, Chinese Academy of Sciences, Kunming, 650201 People’s Republic of China; 2 University of Chinese Academy of Sciences, Beijing, 100049 People’s Republic of China; 3 Lanzhou University of Technology, Lanzhou, 730050 People’s Republic of China

**Keywords:** Metabolomics, Natural products, Metabolism, Bioactivity, Toxicity

## Abstract

**Abstract:**

LC–MS-based metabolomics could have a major impact in the study of natural products, especially in its metabolism, toxicity and activity. This review highlights recent applications of metabolomics approach in the study of metabolites and toxicity of natural products, and the understanding of their effects on various diseases. Metabolomics has been employed to study the in vitro and in vivo metabolism of natural compounds, such as osthole, dehydrodiisoeugenol, and myrislignan. The pharmacological effects of natural compounds and extracts were determined using metabolomics technology combined with diseases models in animal, including osthole and nutmeg extracts. It has been demonstrated that metabolomics is a powerful technology for the investigation of xenobiotics-induced toxicity. The metabolism of triptolide and its hepatotoxicity were discussed. LC–MS-based metabolomics has a great potential in the druggability of natural products. The application of metabolomics should be broadened in the field of natural products in the future.

**Graphical Abstract:**

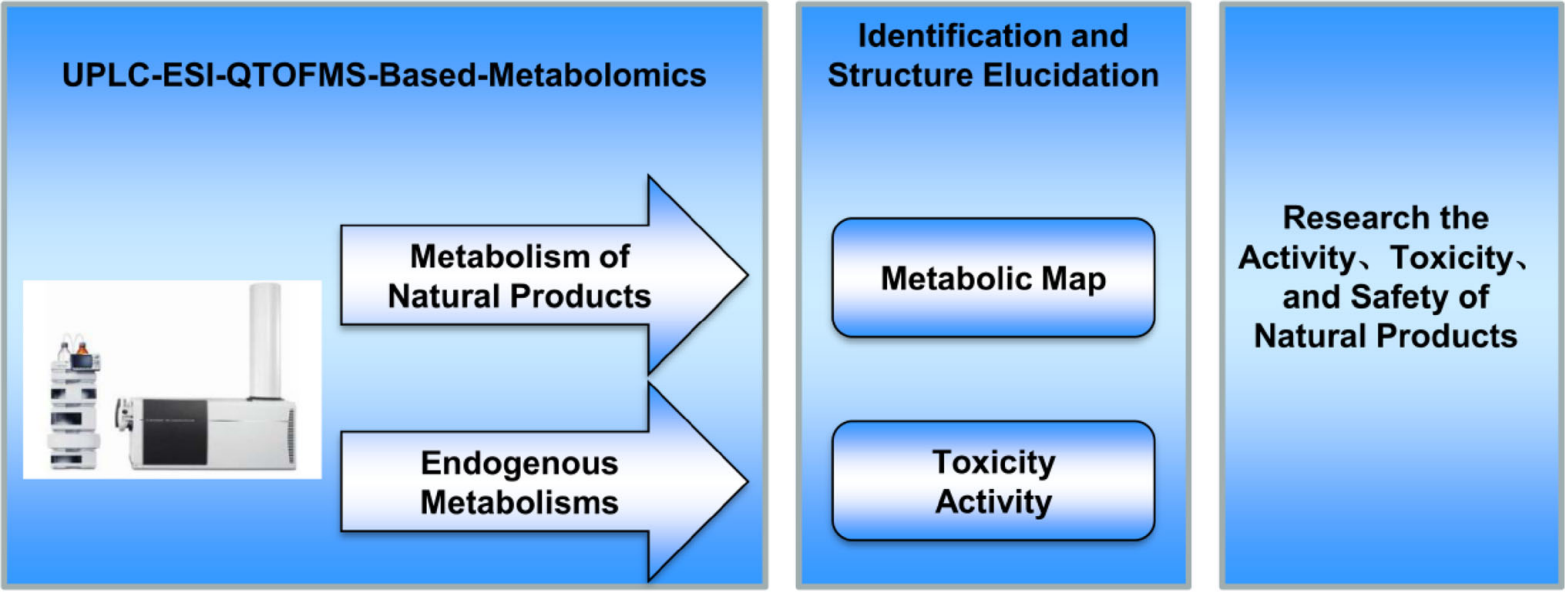

## Introduction

Compounds which are derived from natural sources, e.g., plants, animals and microorganisms, are defined as natural products [1]. Many natural products show the pharmacological or biological activities. Furthermore, natural products are also an important source of inspiration for development of potential novel drugs [2]. Therefore, understanding the activity mechanism of natural products is necessary. In the past few decades, the activity mechanism of natural products has witnessed extensive study in application of modern technology, including metabolomics. However, some natural products are harmful to mammals in spite of their pharmacological or biological activities, such as triptolide (TP). Therefore, understanding the toxicity mechanism of natural products is necessary for their clinical applications.

Metabolomics is an area of investigation that measures changes in small molecules downstream of the genome and proteome, which captures the terminal alteration of endogenous metabolites [3]. The most common platforms used for metabolomics include gas chromatography-mass spectrometer (GC–MS), liquid chromatograph-mass spectrometer (LC–MS), and nuclear magnetic resonance (NMR). Each platform shows its advantages and disadvantages [4]. Compared with the complicated sample preparation (e.g., derivatization) of GC–MS and limited sensitivity of ^1^H NMR, ease of sample preparation and high sensitivity make LC–MS among the most widely used platforms in metabolomics. In the past few years, metabolomics has been used to study natural products metabolic map and natural products-induced activity and toxicity [5]. Firstly, metabolomics can be used to identify metabolic map derived from natural products, which can unbiased determination of metabolites in biofluids (serum, bile, and urine), or extracts from tissues or excreta (feces). Secondary, metabolomics has been successfully introduced into the activity and toxicity studies of natural products. A large number of studies have been performed employing metabolomics to study the activity and toxicity of natural products.

## Metabolic Profile of Nature Products

**Fig. 1:**
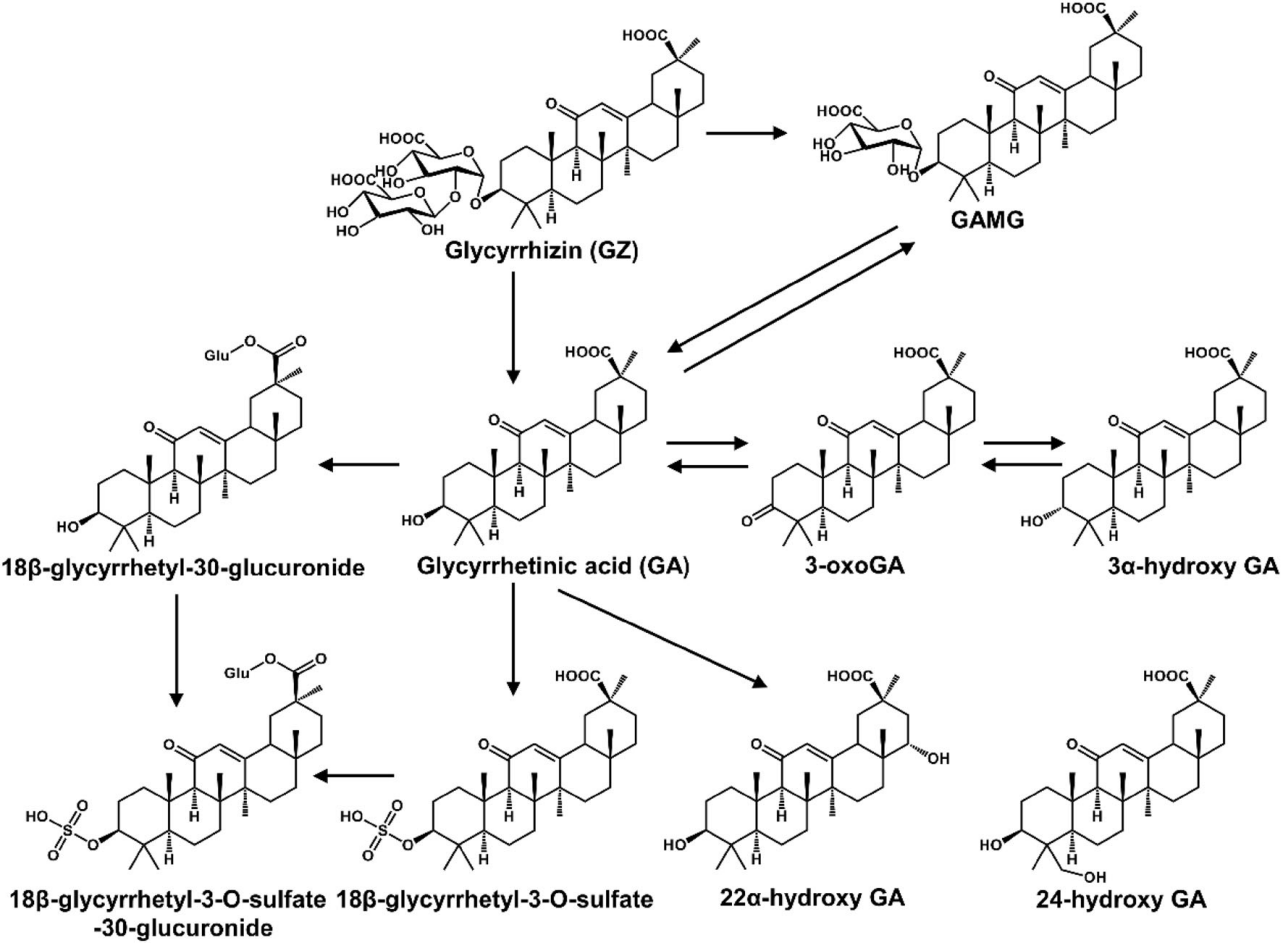
Metabolic map of glycyrrhizic acid. Reproduced with permission from [8]

Humans were frequently exposed to various nature products (e.g., drugs, herbs, and foods). Elucidation of their metabolic pathways would help to determine the efficiency and safety. First, natural products could form pharmacologically inactive metabolites. Second, natural products could form reactive metabolites which induced adverse effects. Third, in some cases, natural products could form active metabolites which increased their therapeutic potential. For example, hydroxyl metabolites of osthole can promote osteoblast growth, which displayed similar activity with osthole [6]. One of the metabolites of [6] -shogaol, (1E,4E)-1-(4′-hydroxy-3′-methoxyphenyl)- deca-1,4-dien-3-one, was tested for cancer cell growth inhibition, which displayed greater activity than [6] -shogaol [7]. Glycyrrhizic acid could be hydrolyzed into glycyrrhetinic acid after oral administration. Both had various pharmacological activities and were widely used in the clinic (Fig. 1) [8]. Seven siamenoside I metabolites, mogroside IVA, mogroside IVE, mogroside III, mogroside IIE, mogroside IA_1_, mogroside IE_1_, and mogrol, can inhibit the induction of Epstein-Barr virus early antigen [9]. Two siamenoside I metabolites, mogroside IVE and mogroside IIIE, can inhibit maltase [9]. A berberine metabolite, dihydroberberine, showed five–ten  folds higher absorption than berberine in the intestine [10]. Three metabolites of hydrastine (M1, M36, and M41) had longer elimination half-lives than hydrastine, which might be better candidates for eliciting the observed effect on CYP2D6 and CYP3A4 activities [11].

**Fig. 2:**
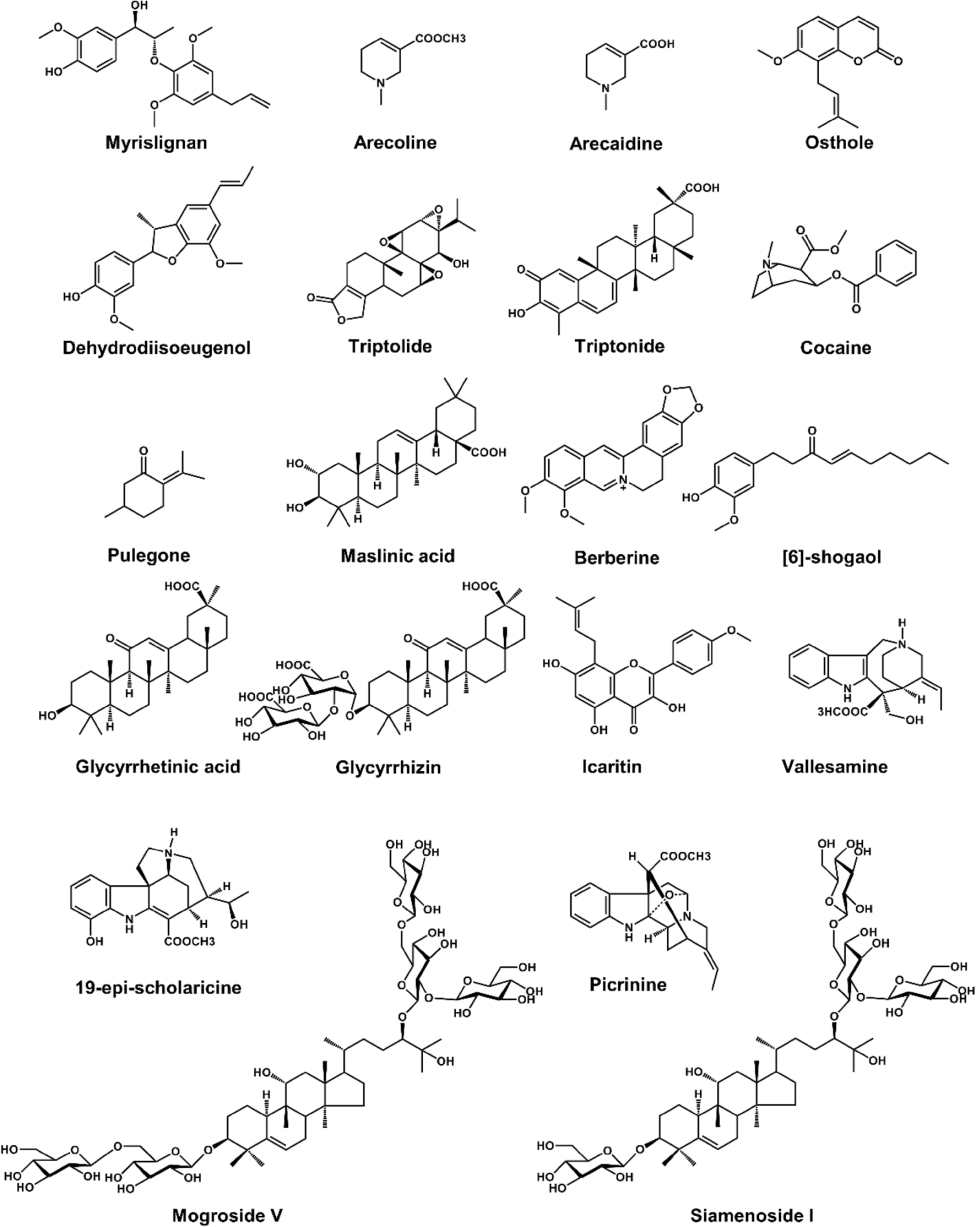
Chemical structure of some natural products. The natural products’ metabolic map has been evaluated by using metabolomics

Liver microsomes (e.g., mouse liver microsome (MLM), human liver microsome (HLM), and recombinant cytochrome P450s (CYPs)) and trapping reagents (e.g., glutathione (GSH) and *N*-acetylcysteine) were used to evaluate the metabolic activation of natural products in vitro. However, in vivo metabolism was the most relevant means to determine the fate of natural products in mammals. Metabolomics was used to study metabolism of natural products nowadays. Using this powerful technology, the metabolic maps of many bioactive natural products have been described, such as myrislignan (MRL) [12], acecoline [13], osthole [14], dehydrodiisoeugenol (DDIE) [15], TP [5], triptonide (TN) [5], cocaine [16, 17], pulegong [18], maslinic acid [19], berberine [20], [6] -shogaol [7], glycyrrhetinic acid [8, 21–23], glycyrrhizic acid [8, 21], lcaritin [24], mogroside V [25], siamenoside I [9], vallesamine [26], 19-epi-scholaricine [26], and picrinine [26] (Table 1 and Fig. 2).

**Table 1:** List of metabolomics application in the study of natural products

Compound/extract	Source	Key findings	References
Metabolic map			
Myrislignan	*Myristica fragrans* Houtt.	19 Novel metabolites	[12]
Arecoline	*Areca catechu* L.	11 Metabolites	[13]
Arecaidine	*Areca catechu* L.	6 Metabolites	[13]
Osthole	*Cnidium moonieri* (L.) Cussion/*Angelica pubescens*	23 Novel metabolites	[14]
Dehydrodiisoeugenol	*Myristica fragrans* Houtt.	7 Novel metabolites	[15]
Triptolide	*Tripterygium wilfordii* Hook. f.	8 Novel metabolites	[5]
Triptonide	*Tripterygium wilfordii* Hook. f.	15 Novel metabolites	[5]
Cocaine	*Erythroxylum novogranatense* (Morris) Hier.	Differential metabolic pathway in mouse and rat	[16]
Pulegone	*Nepeta piperita*	GSH conjugated pulegone metabolites	[18]
Berberine	Goldenseal	3 Novel metabolites	[20]
Maslinic acid	*Olea europaea L.*	7 Metabolites	[19]
[6]-shogaol	*Zingiber officinale*	5 Metabolites were found in mouse, rat, dog, monkey, and human	[7]
Glycyrrhetinic acid	Liquorice root	Glycyrrhetinic acid can affect the activities of CYPs, UGTs and P-gp, and is major metabolized by CYP3A4	[8, 21–23]
Glycyrrhizic acid	Liquorice root	Glycyrrhizic acid can affect the activities of CYPs, UGTs and P-gp	[8, 21]
Icaritin	*Epimedium*	3 Metabolites	[24]
Mogroside V	Siraitiae Fructus	77 New metabolites	[25]
Mogroside V	Siraitiae Fructus	23 and 26 Metabolites were observed in healthy and T2D model rats	[59]
Siamenoside I	Siraitiae Fructus	23 New metabolites	[9]
Vallesamine	*Alstonia scholaris*	6 Metabolites	[26]
19-epi-scholaricine	*Alstonia scholaris*	4 Metabolites	[26]
Picrinine	*Alstonia scholaris*	11 Metabolites	[26]
Toxicity			
Noscapine	Opium	Noscapine generates reactive metabolites	[50]
Bupleurotoxin	*Bupleurum longiradiatum*	Bupleurotoxin induces cerebral lesion	[48]
Triptolide	*Tripterygium wilfordii* Hook. f.	The hydroxyl group at C-14 is responsible for triptolide hepatotoxicity	[5]
Celastrol	Thunder of God Vine	Uridine deficiency contributes to mitochondrial apoptosis induced by celastrol	[52]
*Annona aquamosa*	*Annona aquamosa*	20 Endogenous metabolites are identified to predict its hepatotoxicity and nephrotoxicity	[60]
*Ariseammatic Rhizoma*	*Arisaema erubescens* (Wall.) Schott	*Ariseammatic Rhizoma* induced nephrotoxicity	[49]
*Butea monosperma*	*Butea monosperma*	The safety of *Butea monosperma* can be used as potential anti-diabetic phytopharmaceuticals	[51]
*Euphorbia fischeriana* Steud	*Euphorbia fischeriana* Steud	Metabolic profiles contribute to a better understanding of its adverse effects	[61]
Polygoni Multiflori Radix	*Polygonum multiflorum* Thunb.	Polygoni Multiflori Radix induces hepatotoxicity	[47]
Cocaine	*Erythroxylum novogranatense* (Morris) Hier.	Disrupt amino acid and fatty acid metabolism	[17, 62]
Activity			
Schisandrol B	*Schisandra sphenanthera*	Schisandrol B protects lithocholic acid-induced cholestasis	[63]
Osthole	*Cnidium moonieri* (L.) Cussion/*Angelica pubescens*	Lipid-lowering potential	[5]
Dehydrodiisoeugenol	*Myristica fragrans* Houtt.	Anti-bacterial potential	[15]
Oxyphylla A	*Alpinia oxyphylla*	Oxyphylla A protects neurotoxicity in vitro and in vivo	[64]
Berberine	*Rhizoma Coptidis*	The anti-diabetic effect of berberine	[65, 66]
Anthricin	*Chameacyparis obtusa*	Anthricin protects against human colorectal cancer cell line	[67]
Fenugreek Galactomannan	*Trigonella foenum*-*graecum* L.	Fenugreek galactomannan protects diabetic hyperglycemia	[68]
Glaucocalyxin A	*Rabdosia japonica.*	Glaucocalyxin A has antitumor activity	[69]
Gypenoside	*Gynostemma pentaphyllum* (Thunb.) Makino.	Gypenoside protects tetrachloride-induced liver fibrosis	[70]
Resveratrol	*Smilax china* L.	Resveratrol is an effective quorum sensing inhibitor against *Pseudomonas aeruginosa* PAO1	[71]
Puerarin	*Pueraria lobate* (Willd.) Ohwi	Puerarin ameliorates effects on blood stasis	[72]
Scutellarin	Erigeron breviscapus (vant.) Hand. Mazz	Scutellarin protects ischemic insult	[73]
Nutmeg extract	*Myristica fragrans* Houtt.	Nutmeg protects thioacetamide-induced hepatotoxicity and modulates colon cancer	[41, 45]
*Angelica sinensis*	*Angelica sinensis*	*Angelica sinensis* enriches and regulates the blood	[74, 75]
*Artemisia Capillaris*	*Artemisia Capillaris*	*Artemisia Capillaris* protects fatty liver in diabetic mice	[76]
*Alisma Rhizome*	*Alisma Rhizome*	*Alisma Rhizome* protects fatty liver in diabetic mice	[76]
*Ginkgo biloba* L.	*Ginkgo biloba L.*	*Ginkgo biloba* L. protects myocardial ischemia and positive acceleration exposure	[77, 78]
Fermented black tea	Black tea	Fermented black tea has bacterial inhibitory effect	[79]
*Curcuma longa*	*Curcuma longa*	*Curcuma longa* protects against unbalanced diet and shows its antioxidant activity	[80, 81]
*Dipsacus asper* Wall. ex C.B. Clarke	*Dipsacus asper* Wall. ex C.B. Clarke	*Dipsacus asper* Wall. ex C.B. Clarke protects estrogen deficiency	[82]
Diterpene ginkgolides	*Ginkgo biloba*	Diterpene ginkgolides has antidepressant-like activities in mice	[83]
*Eurycoma longifolia*	*Eurycoma longifolia*	This method might be a new alternative applicable to the fertility assessment	[84]
Epimedium	Epimedium	Epimedium protects glucocorticoid-induced osteoporosis	[85]
Huangqi injection	Astragali Radix	Huangqi injection protects the leucopenia	[86]
Gancao	*Glycyrrhiza uralensis* Fisch.	Gancao protects Fuzi-induced toxicity and its anti-inflammation effect	[87, 88]
*Momordica charantia* L.	*Momordica charantia* L.	*Momordica charantia* L. regulates obesity	[89]
*Morinda citrifolia* L.	*Morinda citrifolia* L.	*Morinda citrifolia* L. regulates obesity	[90]
*Muntingia calabura*	*Muntingia calabura*	*Muntingia calabura* protects CCl_4_-induced liver injury	[91]
*Orthosiphon stamineus*	*Orthosiphon stamineus*	*Orthosiphon stamineus* protects crystal-induced kidney injury	[92]
*Panax notoginseng* Saponins	*Panax notoginseng*	*Panax notoginseng* Saponins protects alcoholic liver injury	[93]

### Metabolic Map of Osthole

**Fig. 3:**
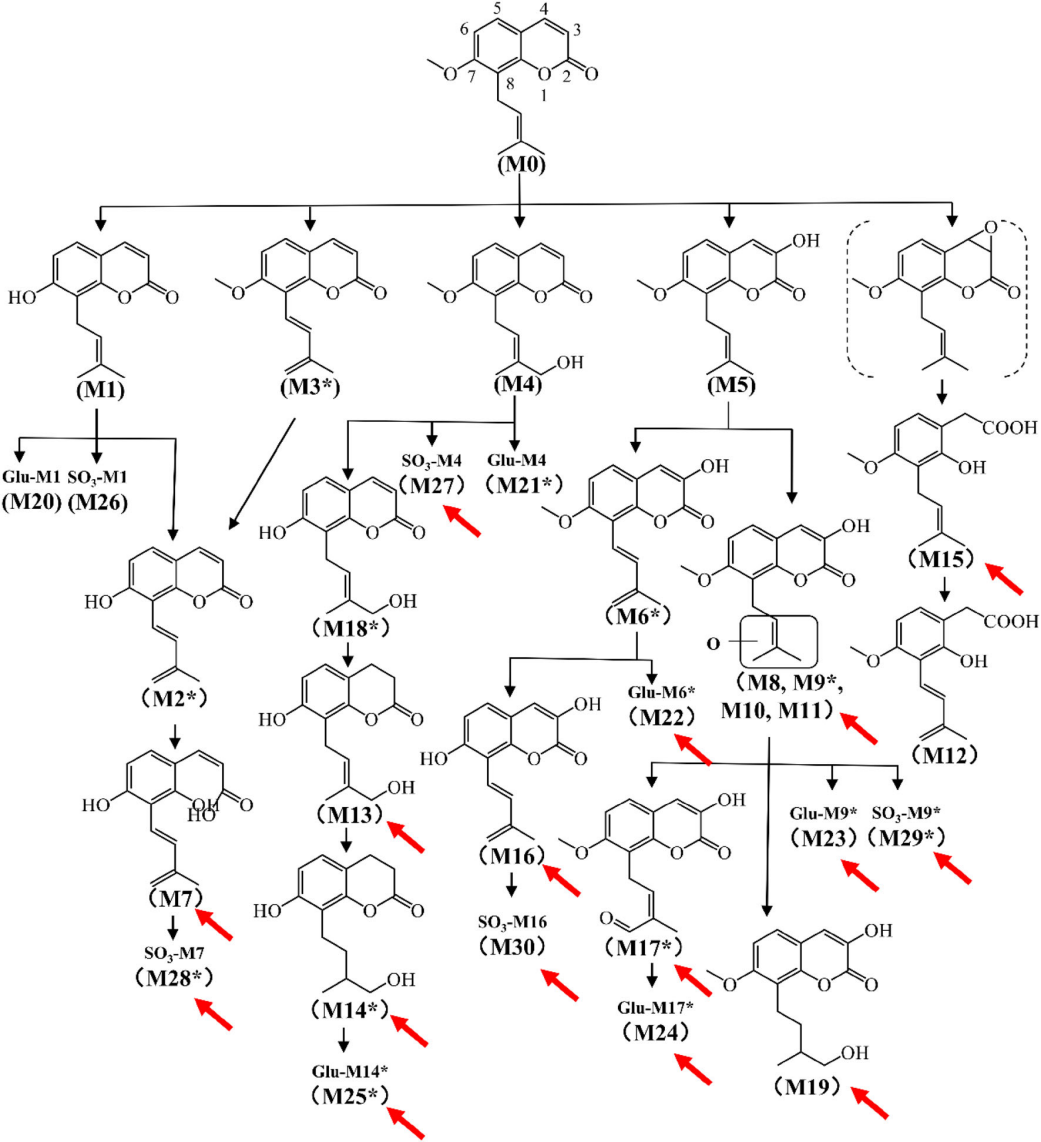
Metabolic map of osthole by using metabolomics. Metabolites marked with asterisk (*) represent isomers were observed. Novel metabolites discovered by UPLC-ESI-QTOFMS-based metabolomics were shown by red arrows. Reproduced with permission from [14]

Osthole is widely distributed in *Angelica pubescens* and *Cnidium moonieri* (L.) Cussion, which shows therapeutic effects on hyperglycemia [27], non-alcohol fatty liver disease [28], and cancers [29]. Recently, the complete metabolic profiling of osthole was elucidated using ultra-performance chromatography electrospray ionization quadrupole time-of-flight mass spectrometry (UPLC-ESI-QTOFMS)-based metabolomics. Forty-one osthole metabolites were identified and structurally elucidated in vitro and in vivo, of which twenty-three were novel metabolites (Fig. 3). Novel metabolites discovered by UPLC-ESI-QTOFMS-based metabolomics were shown by red arrows (Fig. 3). CYPs screening showed that CYP3A4 and CYP3A5 were the primary enzymes contributing to the metabolism of osthole [5]. Hydroxylation, hydrogenation, demethylation, dehydrogenation, glucuronidation, and sulfation were the major metabolic pathways for the metabolism of osthole [5].

### Metabolism Map of DDIE

DDIE is a major benzofuran-type neolignane in *Myristica fragrans* Houtt., which shows various biological activities, including inhibition of hepatic drug metabolism enzyme [30] and anti-bacterial activities [31]. Metabolomics revealed that a total thirteen DDIE metabolites were characterized, including seven novel metabolites [15]. Recombinant CYPs screening showed that CYP1A1 were the primary enzymes contributing to the metabolism of DDIE [15]. In addition, demethylation and ring-opening reaction were the major metabolic pathways for the metabolism of DDIE [15].

### Metabolism Map of MRL

**Fig. 4:**
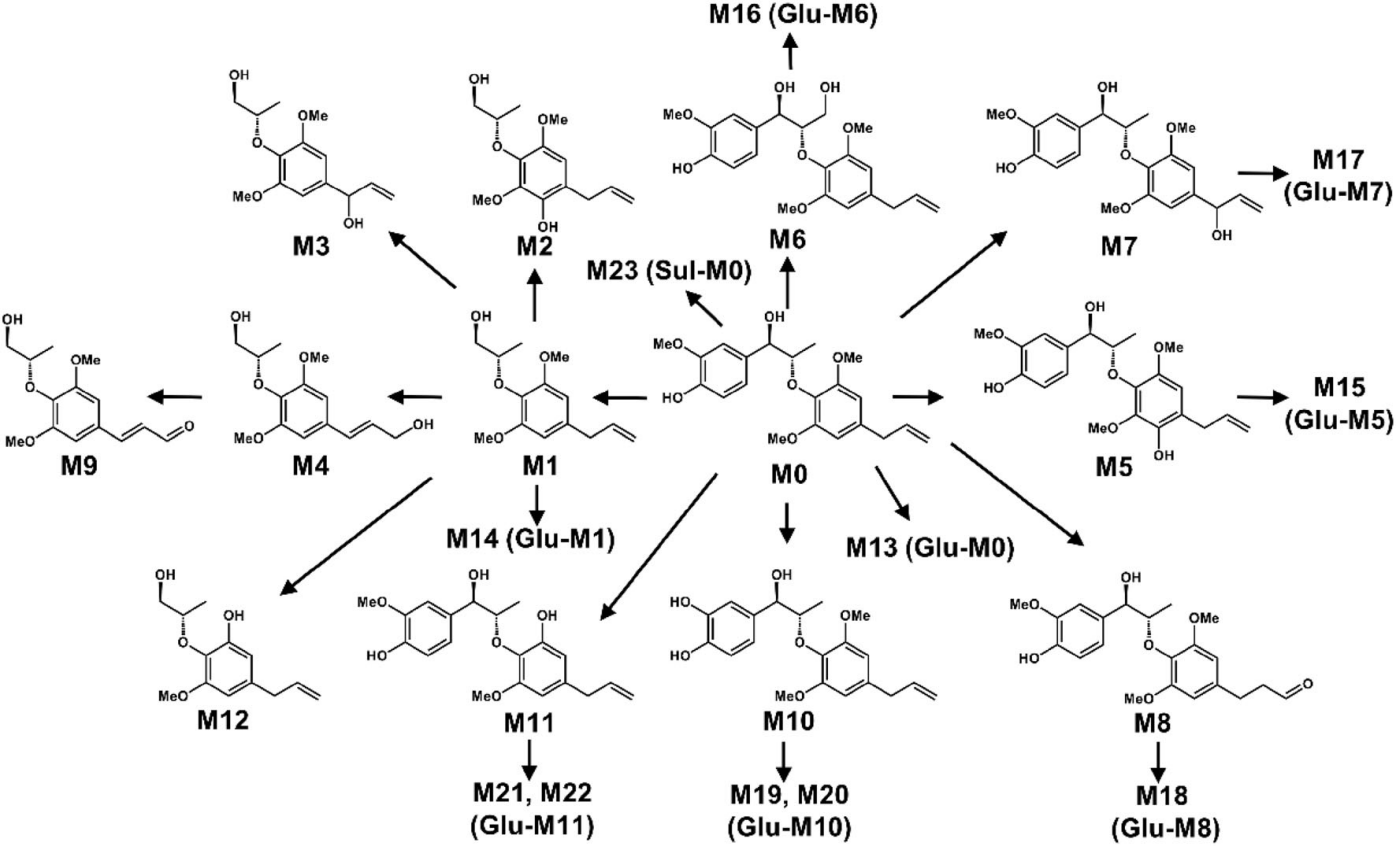
Metabolic map of myrislignan by using metabolomics. Reproduced with permission from [12]

MRL is a bioactive 8-*O*-4′-neolignan in *Myristica fragrans* Houtt., which can decrease the production of nitric oxide induced by lipopolysaccharide [32]. UPLC-ESI-QTOFMS-based metabolomics revealed that a total of twenty-three MRL metabolites (nineteen were newly identified) were determined in both in vivo and in vitro [12] (Fig. 4). Hydroxylation and demethylation were the major metabolic pathways in vitro and in vivo, respectively. Recombinant CYPs screening showed that CYP3A4 and CYP3A5 were the primary enzymes responsible for the metabolism of MRL [12]. These results provided important information for the metabolism of 8-*O*-4′-neolignans in *Myristica fragrans* Houtt. [12].

## Metabolomics Predicts the Pharmacological Effects of Natural Products

### Biological Activity of Osthole

**Fig. 5:**
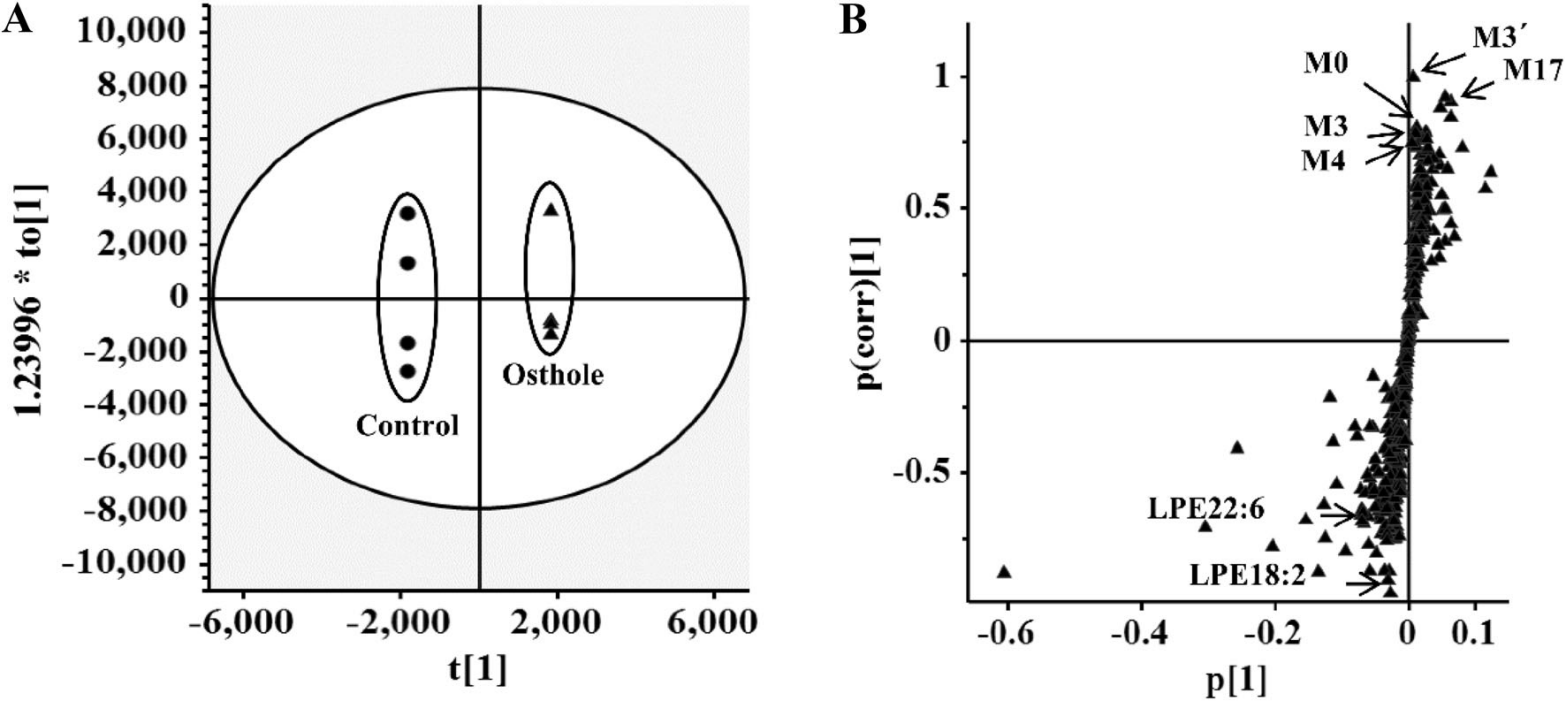
Analysis of osthole-treated mouse plasma and control group in the positive mode in OPLS-DA score plots (**a**) and *S*-plot (**b**). Reproduced with permission from [14]

Earlier studies reported the protective effects of osthole on various metabolic diseases, such as hyperlipidemic and fatty liver [28, 33]. To determine the potential endogenous metabolites influenced by 40 mg/kg osthole treatment, the mouse serum was analyzed by the approach of UPLC-ESI-QTOFMS-based metabolomics. In the loading scatter *S*-plot of orthogonal projection to latent structures-discriminant analysis (OPLS-DA), two endogenous metabolites, lysophosphatidylethanolamine (LPE) 18:2 and LPE22:6, contributed to the separation of osthole-treated group from vehicle group (Fig. 5). Further targeted metabolomics found that more lysophosphatidylcholines (LPCs) and LPEs were significantly decreased after 3 and 24 h treatment of 40 mg/kg osthole [14]. These results indicated that osthole could cause the decrease of plasma LPCs and LPEs levels, which might be involved in its potential benefit effects on adipogenesis [14].

### Biological Activity of DDIE

**Fig. 6:**
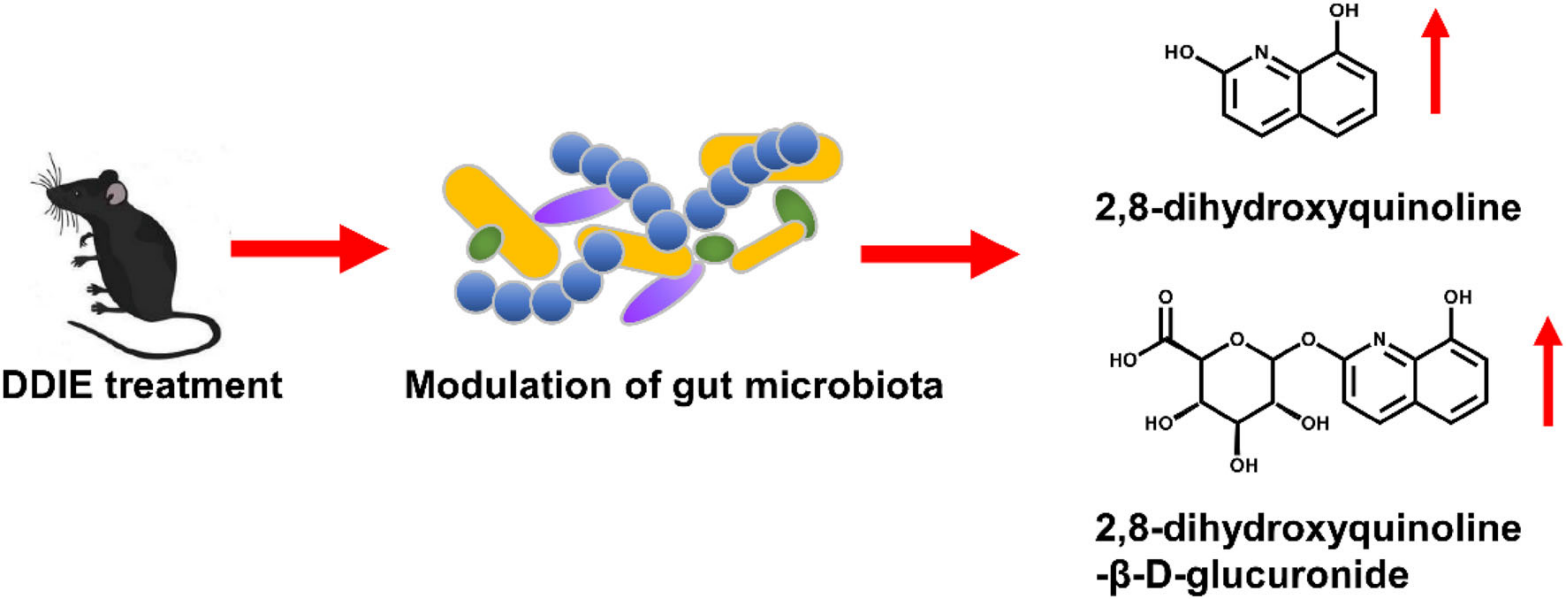
Dehydrodiisoeugenol modulates gut microbiota

DDIE exhibited various anti-bacterial activity in previous study [31]. The levels of endogenous metabolites that were affected by DDIE treatment were examined by UPLC-ESI-QTOFMS-based metabolomics. Two top increased ions, 2,8-dihydroxyquinoline and its glucuronide, were found in the *S*-plot of OPLS-DA [15]. These evidences suggested that DDIE might perform its pharmacological effects through regulating gut microbiota, which might contribute to its anti-inflammatory and anti-bacterial activities [15] (Fig. 6). 2,8-Dihydroxyquinoline and its glucuronide were related with gut microbiota. In previous studies, both 2,8-dihydroxyquinoline and its glucuronide were significantly elevated in mouse urine after tempol treatment [34], and tempol can protect against obesity through regulating the composition of gut microbiota [35]. Furthermore, 2,8-dihydroxyquinoline glucuronide could be detected only in conventional mouse serum, but it could not be found in germ-free mice [36].

## Metabolomics Reveals the Function of Natural Products

### Effect of Nutmeg on Colon Cancer

**Fig. 7:**
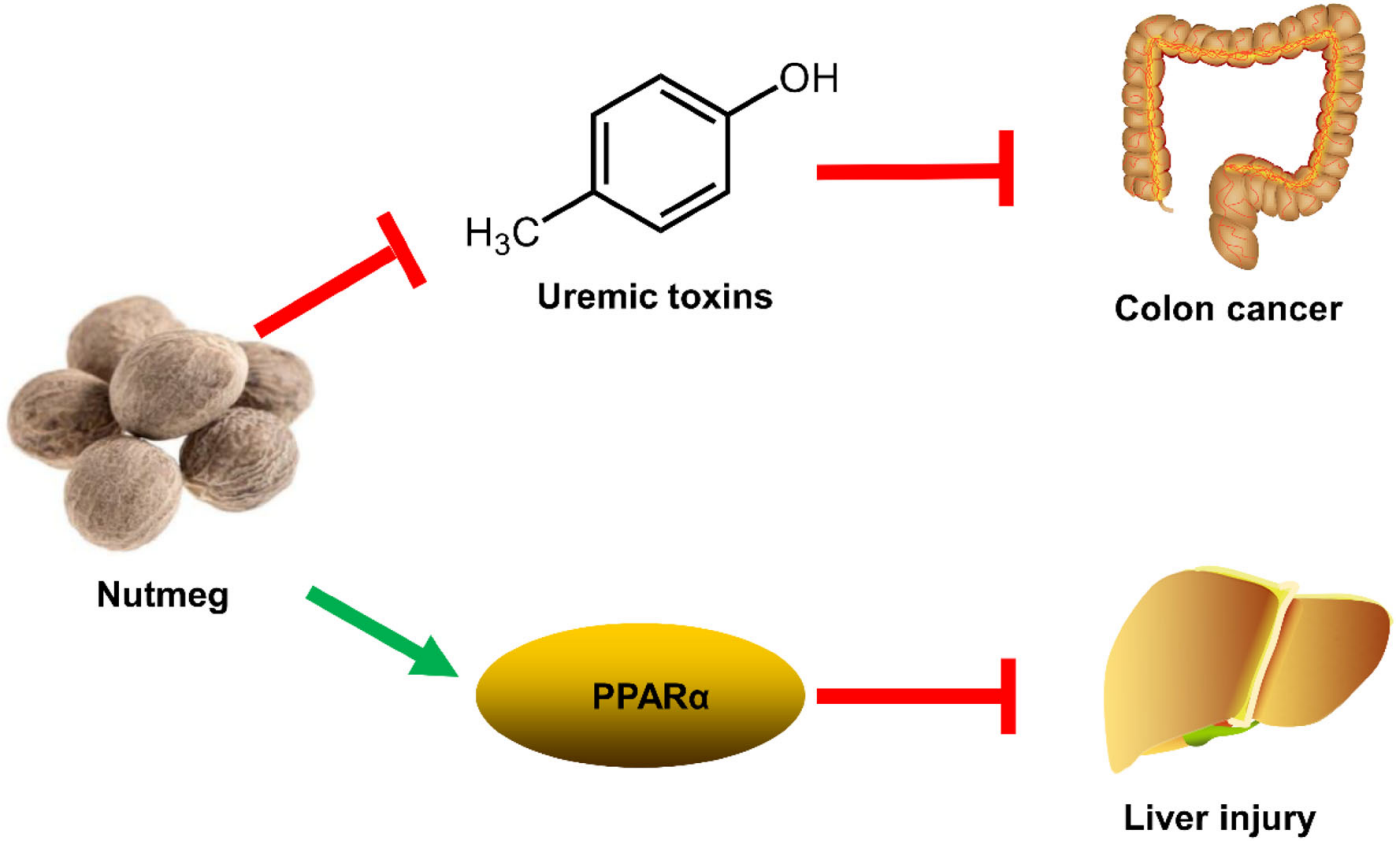
Modulation of colon cancer and liver injury by nutmeg

Nutmeg is the seed of *Myristica fragrans* Houtt., which shows various therapeutic effect in gastrointestinal disorders [37]. In previous study, nutmeg could protect against dextran sulfate sodium-induced colitis in mice [38]. Furthermore, nutmeg was known to exhibit anti-microbial activity against *Helicobacter pylori* and *Escherichia coli* [39, 40]. These results provided a hint that nutmeg might prevent colon cancer via its anti-microbial potential. UPLC-ESI-QTOFMS-based metabolomics revealed the accumulation of uremic toxins (such as cresol sulfate, cresol glucuronide, indoxyl sulfate, and phenyl sulfate) in colon cancer [41]. Uremic toxins could be generated by the disorder of gut microbiota, and it was associated with the elevated interleukin-6 (IL-6) levels [41]. Anti-microbial nutmeg treatment attenuated the levels of uremic toxins and decreased IL-6 levels in colon cancer (Fig. 7). This study suggested that modulation of gut microbiota using nutmeg or other dietary intervention might be effective for the treatment of colon cancer [41].

### Hepatoprotective Effect of Nutmeg

The aqueous extract of nutmeg can protect rats against isoproterenol-induced hepatotoxicity in previous study [42]. Macelignan from *Myristica fragrans* Houtt. can protect cisplatin-induced liver injury through the activation of c-Jun *N*-terminal kinase (JNK) [43]. Furthermore, the antioxidant ability of *Myristica fragrans* Houtt. was related with the inhibition of the lipid peroxidation and superoxide free radicals activity [44]. These results provided a hint that nutmeg might have hepatoprotective activity. A thioacetamide (TAA)-induced acute liver injury in mice was used to explore the mechanism of the protective effects of nutmeg extract. UPLC-ESI-QTOFMS-based metabolomics revealed that treatment with nutmeg led to the recovery of a series of LPCs and acylcarnitines disrupted by TAA exposure [45]. Gene expression analysis demonstrated the protective effect of nutmeg was achieved by the activation of peroxisome proliferator-activated receptor alpha (PPARα). Nutmeg could not protect TAA-induced liver injury in Pparα-null mice, suggesting that its protective effect was dependent on PPARα (Fig. 7) [45]. Furthermore, a neolignane MRL from nutmeg also showed protective effects on TAA-induced liver damage. This study indicated that lignin compounds were the bioactive ingredients of nutmeg [45].

## Metabolomics Explores the Toxicity of Natural Products

**Fig. 8:**
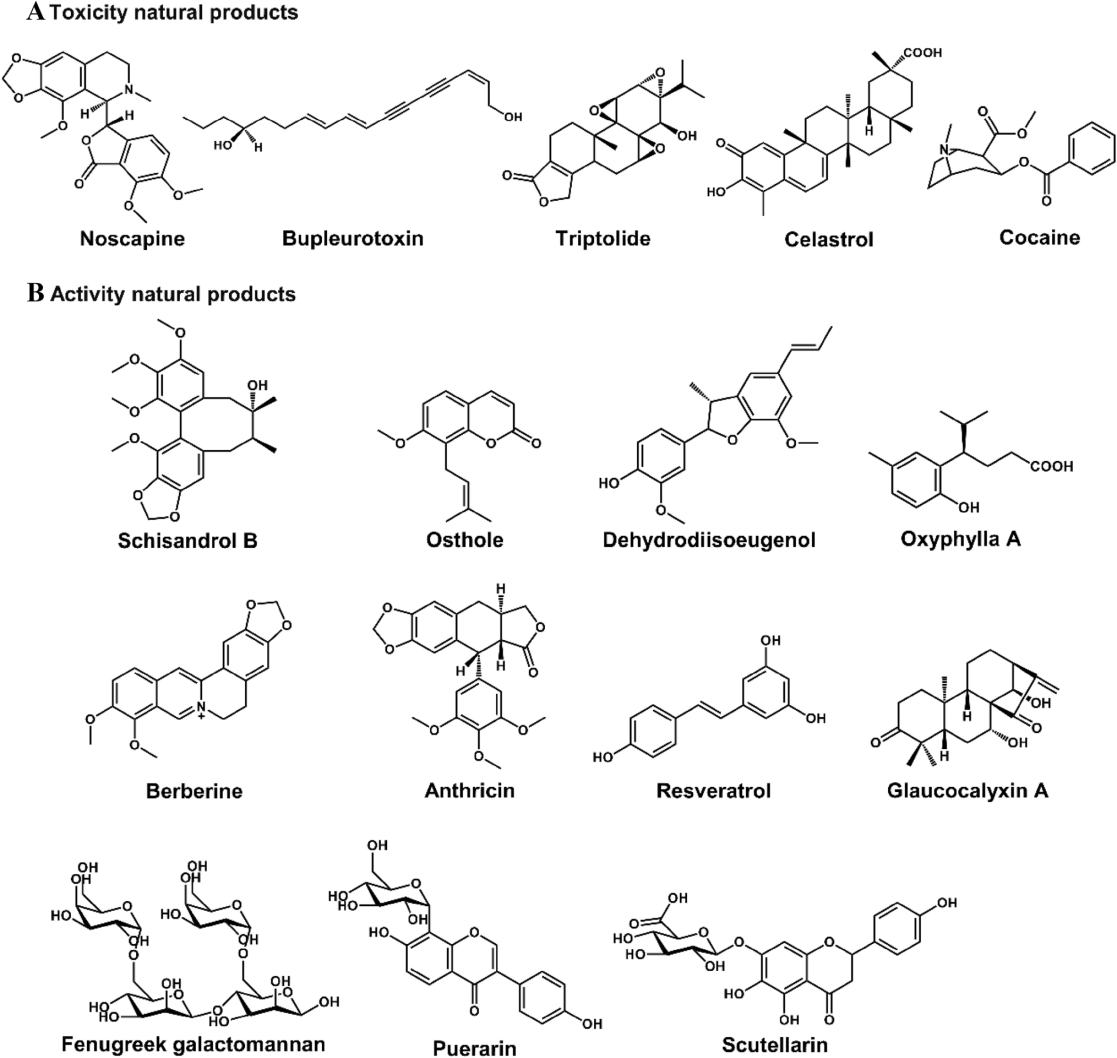
Chemical structures of some natural products. The natural products’ toxicities and activities have been evaluated by using metabolomics

As a key component of systems biology, metabolomics plays an increasingly important role in mechanistic elucidation of the toxicity of natural products, which can be used to determine (I) the toxic metabolisms and (II) the altered endogenous metabolites. (I) The potential toxic metabolisms of nature products include GSH-conjugated metabolites [5] and *N*-oxide metabolites [46]. (II) The altered endogenous metabolites might include bile acids, acylcarnitines, lipids, amino acids, long chain fatty acids, and dicarboxylic acids. Metabolomics can be used to evaluate hepatotoxicity [47], cerebral lesion [48], and nephrotoxicity [49], and evaluate the safety of natural products, such as noscapine and *Butea monosperma* extract (Table 1) [50, 51]. Using the powerful technology, the toxicity and safety of various natural products have been evaluated, such as noscapine [50], bupleurotoxin [48], celastrol [52], TP [5], and various extracts of natural products (Table 1 and Fig. 8).

### Hepatotoxicity of TP

**Fig. 9:**
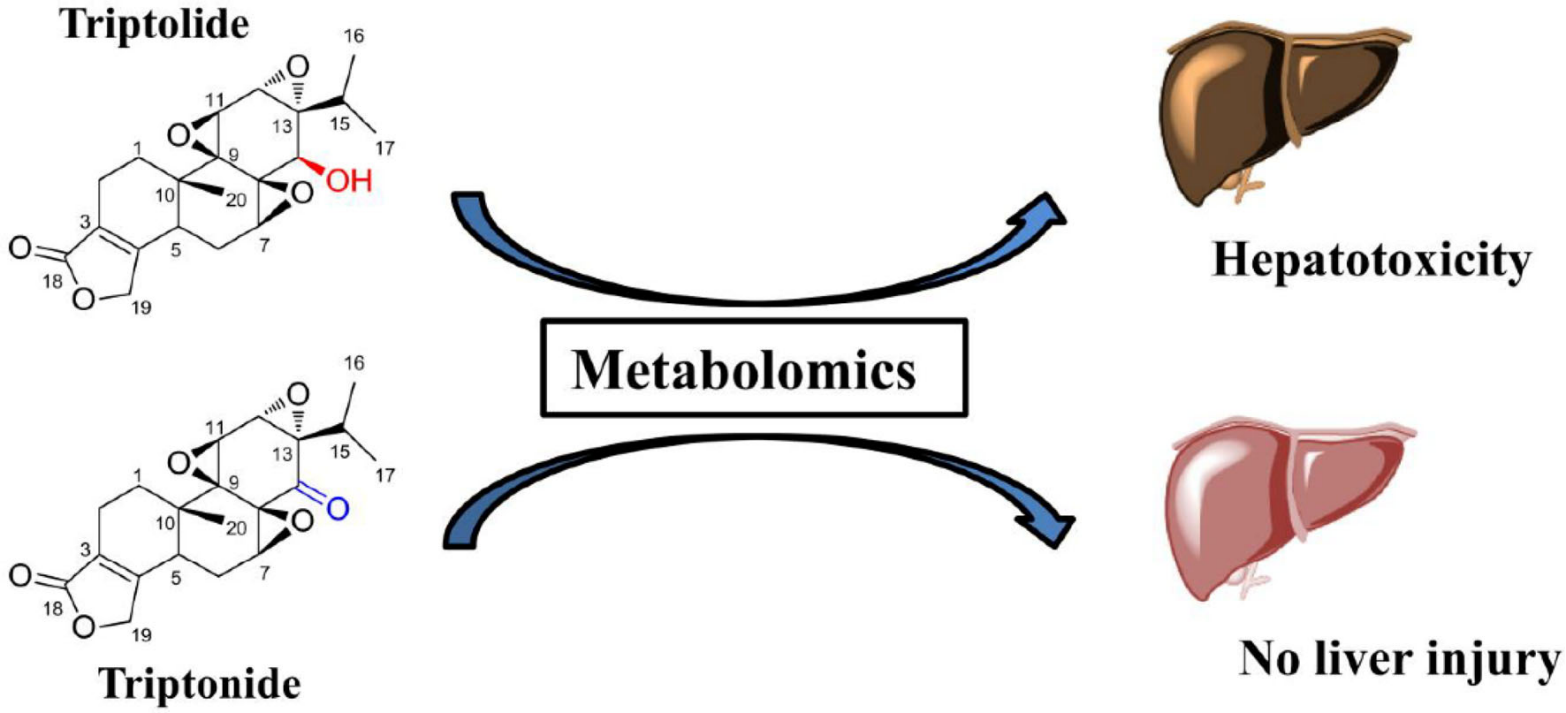
Differential metabolisms of triptolide and triptonide. Reproduced with permission from [5]

TP and TN are two of the main bioactive ingredients isolated from *Tripterygium wilfordii* Hook. f.. Although two compounds showed the similar chemical structures, their toxicities were different. It was reported that severe hepatotoxicity can be induced by TP in animals and humans [53], but the toxicity was not induced by TN in animals [54]. The activities of aspartate transaminase (AST) and alanine transaminase (ALT) in the serum were dramatically increased in TP-treated group at the dose of 1 mg/kg, while AST and ALT levels were not changed in TN-treated group at the same dose [5]. In order to understand the differences of TP- and TN-induced hepatotoxicity, the metabolic pathways of TP and TN were compared in HLM by UPLC-ESI-QTOFMS based metabolomics. Twenty-five drug metabolites were identified by metabolomics for both TP and TN, and eight were found to be novel. Metabolomics showed that although hydroxylation and demethylation were the major metabolic pathways for TP and TN, there were significant metabolic differences between TP and TN [5]. Furthermore, TP showed significantly lower metabolic rate in liver microsome than TN. This study reveals that the hydroxyl group at C-14 in the molecular structure of TP plays an important role in TP-induced hepatotoxicity (Fig. 9) [5].

### Hepatotoxicity of Noscapine

**Fig. 10:**
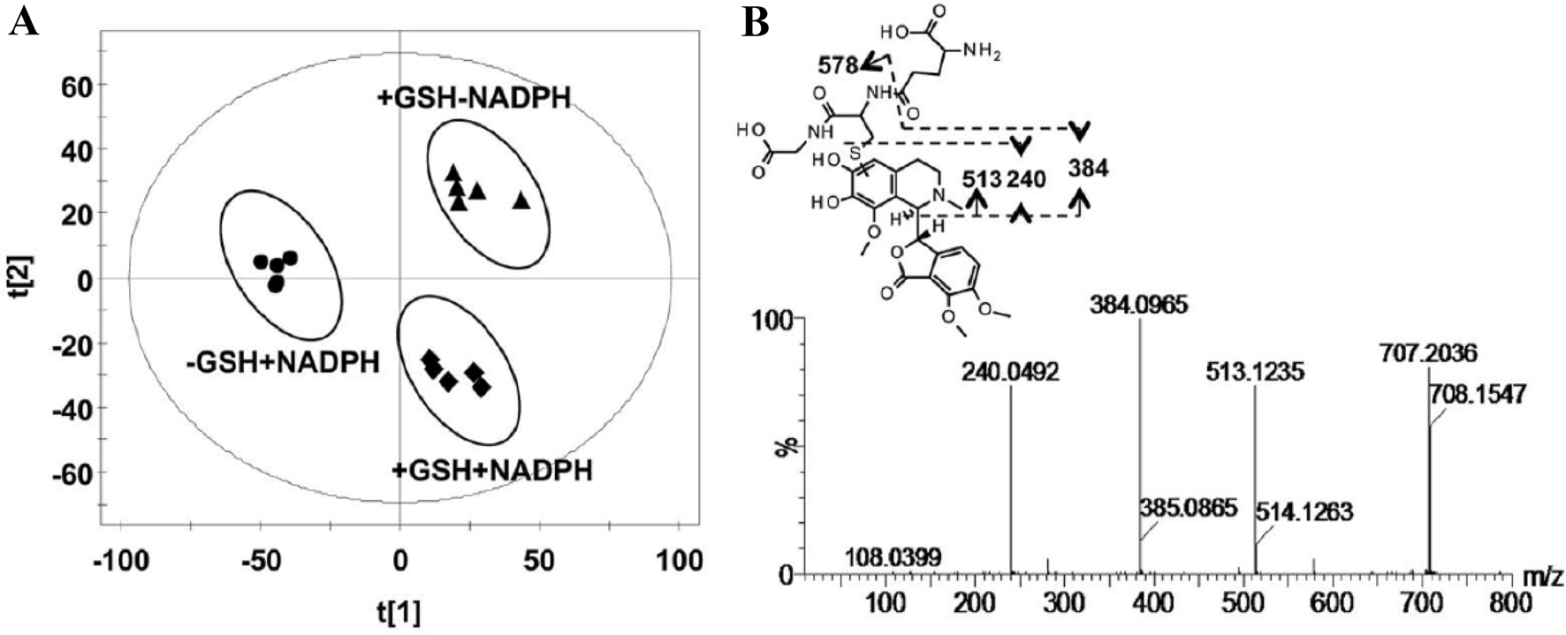
Metabolomic analysis of GSH adducts in the HLM incubation system. **a** Scores scatter plot of OPLS-DA model. **b** The MS/MS fragments and proposed structure of the GSH adduct. Reproduced with permission from [50]

Noscapine is a phthalideisoquinoline alkaloid isolated from opium, which has been clinically used as an efficient cough suppressant [55]. In recent years, the inhibitory potential of noscapine towards the growth of various tumors had been demonstrated using in vitro and in vivo methods, including glioblastoma, colon cancer, and non-small cell lung cancer [56, 57]. The safety of noscapine was still controversial because methylenedioxyphenyl group had been widely accepted as an important structural alert [50]. Some studies suggested that noscapine was potentially carcinogenic [58]. Therefore, the purpose of the present study was to investigate the bioactivation of noscapine. UPLC-ESI-QTOFMS-based metabolomics was used to analyses the in vitro incubation mixtures, urine and feces samples from mice treated with noscapine [50]. In vitro GSH trapping revealed the existence of an *ortho*-quinone reactive intermediate (Fig. 10). However, the reactive intermediate of noscapine was not discovered in vivo. The GSH, AST, ALT, and alkaline phosphatase (ALP) levels obtained from noscapine-treated mice did not show significant alterations. All these results indicated that noscapine did not induce hepatotoxicity in mice. These results provide important information for the development of noscapine for anti-tumor therapy because of its safety [50].

## Conclusions

Metabolomics has been defined as “the comprehensive and quantitative analysis of all metabolites”, which can be used for the study of natural products’ druggability, including the metabolites derived from natural products, metabolic changes-induced by natural products, and toxicity related to natural products exposure. In the past decade, natural products studies have been greatly aided by the using of LC–MS-based metabolomics. In the future, metabolomics can give more valuable information in the research of natural products, especially when combined with isotopes tracers, genetically modified mice, and a systems biology analysis. First, the combination of metabolomics with stable isotope tracers can be successfully used to both study the metabolism of natural products and monitor metabolite flux in vivo and in vitro. Second, genetically modified mice are also valuable tools to understand the role of specific genes in the xenobiotic metabolism, active and toxicology mechanisms of natural products. Furthermore, metabolomics integrating with other omics, such as genomics and proteomics, can serve as an effective tool for investigating the mechanisms of natural products toxicity and activity.

Metabolomics technology is also widely used for drug discovery and development. Importantly for the pharmaceutical industries, the technology has advanced to find hundreds of endogenous and exogenous metabolites in urine, plasma, and tissue extracts. Metabolomics has the potential to make a powerful impact in preclinical drug development studies, including the identification of new targets, the elucidation of the mechanism of action of new drugs, the development of safety and efficacy profiles, as well as the absorption, distribution, metabolism, and excretion (ADME) of new drugs. More importantly, toxicity is a leading cause of attrition at all stages of the drug development process. Metabolic profiling has the potential to identify toxicity early in the drug discovery process, which can save time and money for pharmaceutical companies.
